# A 556 kb deletion in the downstream region of the *PAX6* gene causes familial aniridia and other eye anomalies in a Chinese family

**Published:** 2011-02-10

**Authors:** Fang Cheng, Wulian Song, Yang Kang, Shihui Yu, Huiping Yuan

**Affiliations:** 1 Department of Ophthalmology, the 2nd Affiliated Hospital of Harbin Medical University, Harbin, China; 2 Department of Ophthalmology, the 1st Affiliated Hospital of Harbin Medical University, Harbin, China; 3 Department of Pathology, Children’s Mercy Hospitals and Clinics and University of Missouri-Kansas City School of Medicine, Kansas City, MO

## Abstract

**Purpose:**

The paired box gene 6 (*PAX6*) on human chromosome 11p13 is an essential transcription factor for eye formation in animals. Mutations in *PAX6* can lead to varieties of autosomal-dominant ocular malformations with aniridia as the major clinical signs. Known genetic alterations causing haplo-insufficiency of *PAX6* include nonsense mutations, frame-shift mutations, splicing errors, or genomic deletions. The purpose of this study was to identify genetic defects as the underlying cause of familial aniridia in a large Chinese family.

**Methods:**

All exons of *PAX6* in the proband were sequenced by the Sanger sequencing technique. The genome of the proband was evaluated by a microarray-based comparative genomic hybridization (aCGH). Quantitative real-time PCR was applied to verify the abnormal aCGH findings in the proband and to test five other family members.

**Results:**

There were no detectable pathogenic mutations in the exons of *PAX6* in the proband. The aCGH analysis showed two copies of *PAX6* but revealed a 566 kb hemizygous deletion of chromosome 11p13, including four annotated genes doublecortin domain containing 1 (*DCDC1),* DnaJ homolog subfamily C member 24 (*DNAJC24*)*, *IMP1 inner mitochondrial membrane**(*IMMP1L), *and**elongation factor protein 4 (*ELP4*) downstream of *PAX6*. Quantitative real-time PCR verified the deletion in the proband and further identified the deletion in a blind fashion in four affected family members but not in the one with a normal phenotype.

**Conclusions:**

The 566 kb hemizygous deletion of chromosome 11p13 downstream of *PAX6* should be the cause of the familial aniridia in this Chinese family, although two copies of *PAX6* are intact. aCGH evaluation should be applied if there is a negative result for the mutation detection of *PAX6* in patients with aniridia.

## Introduction

Aniridia (OMIM 106210) is a congenital eye disorder characterized by the complete or partial absence of the iris and iris hypoplasia. Eight-five percent of individuals with aniridia inherit this disorder as an autosomal-dominant trait, 13% occur as part of the autosomal-dominant WAGR syndrome (Wilms’ tumor, aniridia, genitourinary abnormalities, and mental retardation), and the remaining 2% occur as part of other disorders, including Peters’ anomaly and Gillespie’s syndrome, in either autosomal-dominant or autosomal-recessive inheritances [1-3].

The paired box gene 6 (*PAX6*) is located on chromosome 11p13 and contains 14 exons encoding a protein (PAX6) with 422 amino acids. PAX6 is a transcriptional factor controlling development of a diversity of organs and tissues (forebrain, pancreas, and ocular tissues, including corneal epithelium, lens, and retina) by recognizing specific DNA sequences of its downstream target genes [4]. Nonsense mutations or deletions of the *PAX6* gene primarily cause aniridia due to its haplo-insufficiency [5], while missense mutations of this gene are associated with a diversity of eye abnormalities through gain-of-function of the mutated protein, such as Peters’ anomaly, corneal dystrophy and opacification, congenital cataracts, glaucoma, and foveal hypoplasia [2,6,7]. Whereas mutations or intragenic deletions of *PAX6* represent the major causes of aniridia, genomic rearrangements involving the downstream region of *PAX6* were identified in patients with aniridia although both copies of *PAX6* are intact in these patients [8-13].

In the present study, we report a genomic microdeletion in the downstream region of *PAX6* in a large Chinese family with familial aniridia and other eye anomalies, using microarray-based comparative genomic hybridization (aCGH) techniques. To our knowledge this is the first case found in an Asian population and is one of few similar cases with this kind of genetic mechanism [9,11,12].

## Methods

### Patients

**Figure 1:**
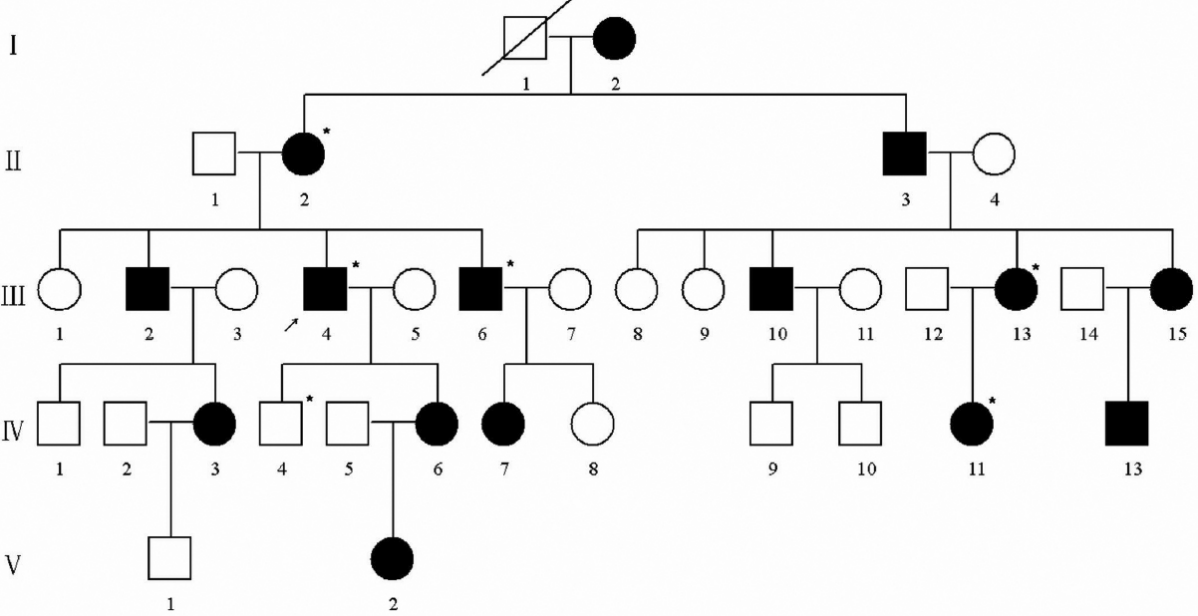
Pedigree of the family in this study. Squares and circles indicated males and females respectively. The symbols in black represent the affected members. The asterisks indicate those subjects who participated in this study. The arrow indicates the proband. The square with a line indicated a deceased individual.

A five-generation Chinese family with familial aniridia was recruited from Heilongjiang province, northeastern China. There were 15 affected individuals in this family from which five affected members (II:2, III:4, III:6, III:13, and IV:11) and one unaffected individual (IV:4) participated in this study (Figure 1). Ocular tests for these six members included visual acuity of naked eyes and corrected visual acuity, which were recorded using the logarithm of the minimum angle of resolution E chart (Precision Vision, Villa Park, IL), slit-lamp biomicroscopy, measurement of intraocular pressure by applanation tonometry, and gonioscopic evaluation of the anterior chamber angle. Systemic evaluation was performed in the six subjects in this study to exclude WAGR syndrome (Wilms’ tumor, aniridia, genitourinary abnormalities, and mental retardation), Axenfeld–Rieger syndrome, iridocorneal endothelial syndromes, sclerocornea, and Peter’s anomaly. Informed consents were obtained from the six individuals in this study. Five affected patients and one family member with normal phenotype were further investigated using molecular techniques. The research protocol for this study was approved by the Harbin Medical University Ethics Committee (Harbin, China).

### Experiments

All exons of *PAX6* in the proband (III:4) were amplified and sequenced with the ABI BigDye Terminator Cycle Sequencing kit v3.1, (ABI Applied Biosystems, Foster City, CA) according to the described protocol [14]. The superimposed mutant PCR products were subcloned into pGEM-T vector (Promega, Madison, WI) and sequenced to identify the mutation. The genomic DNA from the proband was analyzed by aCGH using the Agilent Human Genome Microarray Kit 244K (Agilent Technologies, Santa Clara, CA) based on the published procedures [15]. The digested test DNA and reference DNA were labeled with cyanine (Cy)3-deoxyuridine triphosphate (dUTP) or Cy5-dUTP, respectively. Following purification, the appropriate Cy3-labeled test DNA and Cy5-labeled reference DNA were mixed together and combined with 2× Hybridization buffer (Agilent), 10× blocking agent (Agilent), and Human Cot-1 DNA (Invitrogen, Carlsbad, CA). The hybridization mixture was slowly dispensed to a microarray chip and assembled with an Agilent SureHyb chamber. After washing, all microarray slides were scanned on an Agilent Microarray Scanner G2565BA with 5-μm resolution. Captured images were transformed to data with Feature Extraction Software, version 9.5 (Agilent), and then imported into Agilent CGH Analytics 3.2.5 software for analysis. Quantitative real-time PCR (qPCR) was performed to verify the abnormal aCGH findings in the proband and to test other family members, according to published guidelines [16]. Individual optimization qPCRs were performed in a 20 μl volume including 25 ng template DNA, 200 nM of each primer, and 1× Premix-Choice with ROX reference dye in an initial denaturation of 93 °C for 10min, followed by 35 cycles of 93 °C for 1 min and 61 °C for 1 min, and 72 °C for 1 min. The three sets of primers for qPCR analysis (Table 1) include primers targeted to the elongation factor protein 4 *(ELP4)* gene within the deleted genomic region, primers within the *PAX6* gene, and reference primers within the Oral-facial-digital syndrome 1 protein *(OFD1)* gene on the human X chromosome. Both *ELP4* and *PAX6* were assayed as test genes compared with *OFD1*, and both *ELP4* and *PAX6* were assayed as test/controls.

**Table 1:** Primer information.

**Primer sets**	**Primers sequences**	**Amplified region**	**Amplicon size**	**Target genes**
Target primers	Forward: aatgtttcggcctacgatgggagt	chr11:31586811–31586956	146 bp	*FOXC2*
** **	Reverse: tttagcacccacttacccttccca	** **	** **	** **
Target primers	Forward: tcataaacactgcagccagcctct	chr11:31781362–31781507	146 bp	*PAX6*
** **	Reverse: tcccaacactgcagagaccttgaa	** **	** **	** **
Reference primers	Forward: aggtgttctgctgctgagatggaa	chrX:13693097–13693233	137 bp	*OFD1*
** **	Reverse: tccctttgtgcccagatgaagaga	** **	** **	** **

## Results

### Clinical findings

The clinical findings are summarized in Table 2. The proband (III:4), was diagnosed with bilateral iris coloboma, optic atrophy of the left eye, right corneal opacity, and strabismus. Because of glaucoma, the proband had undergone cyclocryotherapy on the left eye in 2002. Other recurrent symptoms in the other affected participants include optic atrophy, retinitis pigmentosa, cataract, subluxation of lens, and dysplasia of trabecular meshwork, fovea, and optic nerve (Table 2). Poor vision is due to foveal or optic nerve hypoplasia, cataract, glaucoma and amblyopia. No visible autistic problems or intellectual disabilities were identified in these affected individuals.

**Table 2:** The clinical findings of individuals in this study.

** **	** **	** **	**Naked visual acuity**		**IOP (mm Hg)**		** **	** **	**Corrected visual acuity with patients’ own spectacles**		**Anterior chamber angle**	
**Individual**	**Age**	**Sex**	**Right**	**Left**	**Right**	**Left**	**Clinical findings**	**Eye surgery**	**Right**	**Left**	**Right**	**Left**
II:2	74	female	HM/10cm	HM/10cm	17	15	bilateral optic atrophy; iris coloboma; retinitis pigmentosa; pannus and aphakia of both eyes	cataract surgery	HM/10cm	HM/10cm	dysplasia of trabecular meshwork and closure of the trabecular meshwork by residual iris stump	dysplasia of trabecula r meshwork and closure of the trabecular meshwork by residual iris stump
III:4	46	male	NLP	FC/20cm	15	22	optic atrophy of left eye;	cyclocryotheraphy on the left eye; bilateral iris coloboma, corneal opacity and strabismus of right eye	NLP	0.2	Cannot been seen clearly	the rudimentary stump of iris and the angle covered by the iris stump
III:6	43	male	0.04	0.02	22	19	bilateral cataract, iris coloboma and subluxation of lens;	none	0.5	0.4	the rudimentary stump of iris and the angle covered by the iris stump	the rudimentary stump of iris and the angle covered by the iris stump
III:13	38	female	0.1	0.1	18	15	bilateral aniridia; amblyopia;	none	0.6	0.6	dysplasia of trabecular meshwork	dysplasia of trabecular meshwork
IV:4	22	male	1.0	1.0	16	16	normal	none	1.0	1.0	open	open
IV:11	14	female	0.08	0.1	14	15	bilateral aniridia; amblyopia;	none	0.6	0.6	the rudimentary stump of iris and the angle covered by the iris stump	the rudimentary stump of iris and the angle covered by the iris stump

Notes: IOP: intraocular pressure; HM: hand movement; NLP: no light perception; FC: finger count.

### Sequencing results for *PAX6*

All exons of *PAX6* from the six participants of the family were amplified and sequenced using standard techniques. No intragenic point mutation or deletion could be identified (data not shown).

### Microarray-based comparative genomic hybridization findings in the proband

**Figure 2:**
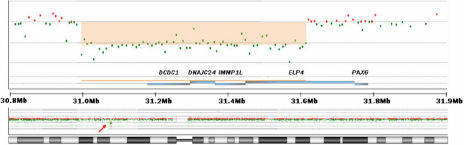
The 556 kb genomic deletion of chromosome 11p13. This deletion harbors four annotated genes, *DCDC1, DNAJC24, IMMP1L*, and *ELP4*.

The aCGH analysis detected 35 copy number variations (CNVs) in the proband’s genome (Table 3). One of them is a 566 kb hemizygous deletion of chromosome 11p13 (chr:31,074,403–31,640,263) with approximately a 123 kb distance from the 3′ end of *PAX6* (chr11:31,762,916–31,796,085) according to HG18 (NCBI 36, March 2006; Figure 2. This deletion contains four annotated genes: doublecortin domain containing 1 (*DCDC1*), DnaJ homolog subfamily C member 24 (*DNAJC24*), IMP1 inner mitochondrial membrane (*IMMP1L*), and elongation factor protein 4 (*ELP4*). The remaining 34 CNVs were considered to be benign because these CNVs were identified in healthy individuals documented in the Database of Genomic Variation or in recent publications [17,18].

**Table 3:** Total CNVs identified in the genome of this individual.

**Number**	**Chromosome**	**Cytoband**	**Start**	**Stop**	**Size**	**Duplication**	**Deletion**	**Gene names**
1	chr1	q21.3	150822873	150848709	25836	2.044245	0	*LCE3C*
2	chr3	p12.3	75895820	75958100	62280	0	−0.682351	*ZNF717*
3	chr3	q26.1	164038717	164101976	63259	0	−0.921074	
4	chr3	q29	196943180	196950295	7115	0	−0.784749	*MUC20, MUC20*
5	chr4	q13.2	69057535	69166014	108479	0	−4.405964	*UGT2B17*
6	chr4	q28.3	131700605	131865354	164749	0	−0.598187	* *
7	chr5	p15.33	802318	878490	76172	0.667749	0	*ZDHHC11*
8	chr5	q13.2	69741118	70422497	681379	0	−1.028287	*GTF2H2C, GTF2H2B, GTF2H2D, SERF1A, SERF1A, SERF1B, SMN1, SMN2, SMN1, SMN2, NAIP, GTF2H2B, GTF2H2C, GTF2H2, GTF2H2D, OCLN, LOC647859*
9	chr6	p22.1	29201691	29260945	59254	0	−1.115529	*OR2J2*
10	chr6	p21.32	32567182	32660290	93108	0	−2.059653	*HLA-DRB5, HLA-DRB6, HLA-DRB1*
11	chr6	p21.32	32595202	32605500	10298	0	−4.758382	*HLA-DRB5*
12	chr6	p12.1	55468168	55501199	33031	0	−0.627628	*HMGCLL1*
13	chr7	p22.3	140013	225040	85027	0.843971	0	* *
14	chr7	q21.2	92484285	92530356	46071	0	−0.870801	* *
15	chr7	q32.3 - q33	132382438	132457449	75011	0	−1.214264	*CHCHD3*
16	chr7	q34	141413152	141438704	25552	1.057004	0	*MGAM*
17	chr7	q34	142158954	142171818	12864	0	−2.550414	*TRY6*
18	chr8	p11.23 - p11.22	39356395	39505456	149061	1.698241	0	*ADAM5P, ADAM3A*
19	chr11	p13	31074403	31640263	565860	0	−1.081172	*DCDC1, DNAJC24, IMMP1L, ELP4*
20	chr11	q11	55124530	55195190	70660	0	−0.554403	*OR4C11, OR4P4, OR4S2, OR4C6*
21	chr12	p13.31	9528390	9585356	56966	1.893183	0	* *
22	chr14	q32.33	105602556	105630289	27733	0	−3.599436	* *
23	chr14	q32.33	105962105	105985658	23553	0.992122	0	* *
24	chr15	q11.2	18809804	18884636	74832	0.536094	0	* *
25	chr15	q11.2	19845742	20080135	234393	0.435061	0	*LOC727924, OR4M2, OR4N4, LOC650137*
26	chr16	p13.3	2638381	2672274	33893	0.722366	0	
27	chr16	p13.11	14955977	15023221	67244	0	−0.94928	*PDXDC1*
28	chr16	q12.2	54354443	54380034	25591	0	−0.650633	*CES4*
29	chr16	q22.1	68706040	68754434	48394	0	−0.495966	*PDPR*
30	chr17	q21.31 - q21.32	41553026	42049740	496714	0	−0.347977	*KIAA1267, LRRC37A, ARL17, ARL17, LRRC37A2, ARL17P1, ARL17P1, ARL17, NSF*
31	chr17	q25.3	77422498	77503885	81387	0.905508	0	*ARHGDIA, THOC4, ANAPC11, ANAPC11, NPB, PCYT2, SIRT7, MAFG, MAFG, LOC92659, PYCR1, PYCR1, MYADML2, NOTUM*
32	chr19	p12	20422376	20493601	71225	0	−0.809494	
33	chr20	p13	1516766	1539355	22589	0	−1.330169	*SIRPB1, SIRPB1*
34	chr22	q12.3	35217849	35225862	8013	0.921708	0	*FOXRED2, FOXRED2*
35	chr22	q13.1	37688858	37715585	26727	0	−0.689394	*APOBEC3A, APOBEC3B*

### Quantitative real-time PCR assays in the proband and other family members

**Figure 3:**
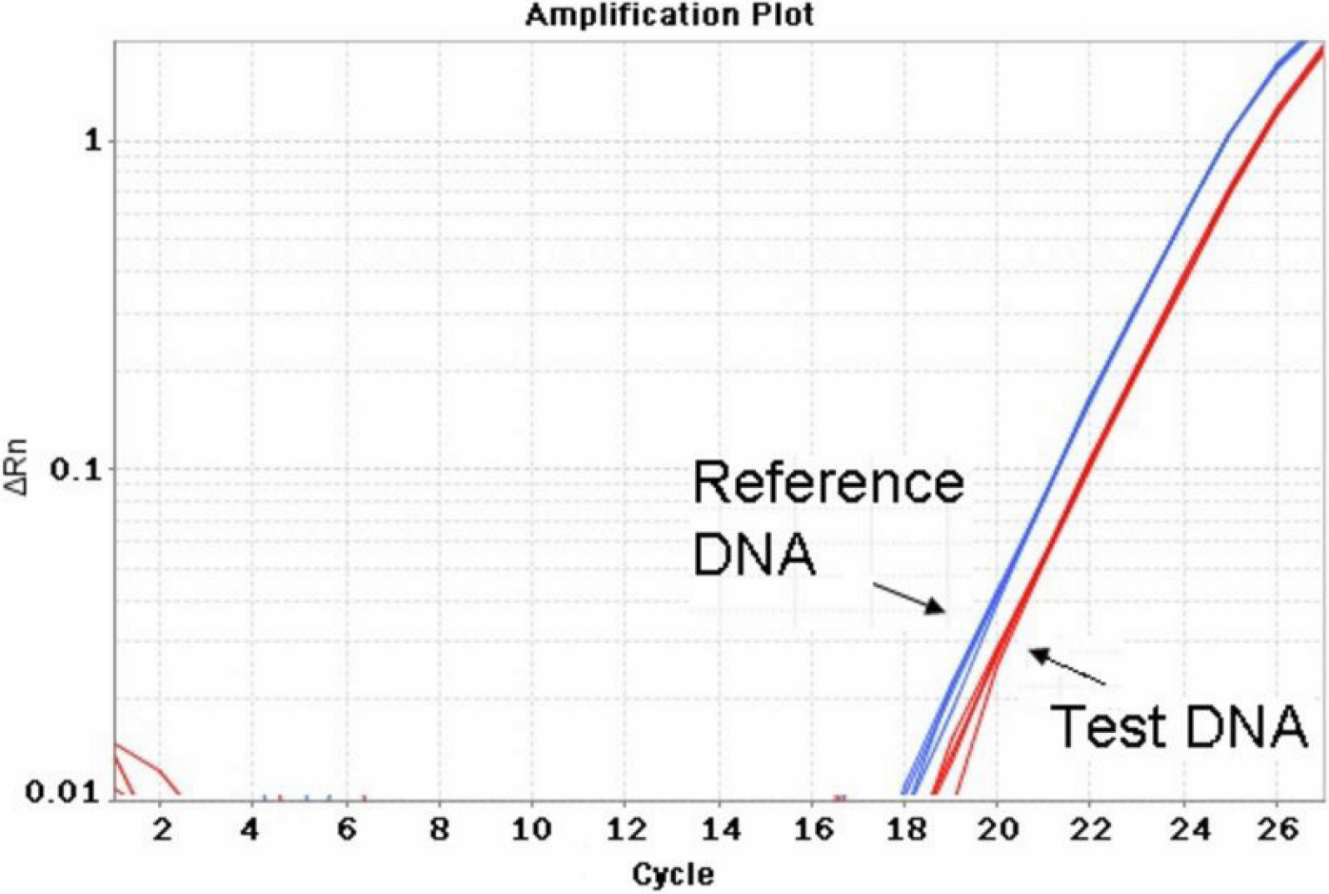
qPCR analysis result. An example of the qPCR amplification plot showed a single copy of the test gene, *ELP4*, in the affected individuals compared to the two copies of this gene in the reference DNA.

The 566 kb hemizygous deletion of chromosome 11p13 in the proband was verified by qPCR using primers targeted to *ELP* within the deleted region, which showed one threshold cycle difference between the patient and reference DNA samples for the amplification of the test gene (Figure 3). Two copies of *PAX6* were confirmed by qPCR, consistent with the aCGH results in this individual (data not shown). We used the qPCR method for testing the other five family members in a blind fashion, four affected family members with eye anomalies, and one healthy individual. All four patients were confirmed to carry the deletion, including *ELP*, but not *PAX6*. The phenotypic normal individual showed two copies for both *ELP* and *PAX6* (data not shown).

## Discussion

The major finding in this Chinese family with familial aniridia is the presence of a 566 kb heterozygous deletion containing four annotated genes: *DCDC1, DNAJC24, IMMP1L,* and *ELP4*. The proximal breakpoint of this deletion is approximately 123 kb from the 3′ end of *PAX6*. We postulate that this 566 kb heterozygous deletion is the underlying cause of the familial aniridia and acts by disrupting the transcription in one of the two PAX6 alleles, even though the two copies of *PAX6* were intact in all individuals investigated in this study.

Our finding provides further evidence of the existence of the remote 3′ regulatory elements in the downstream region of *PAX6* controlling the expression of this gene, if disrupted, leading to aniridia and other eye anomalies. To our knowledge, this is the first case found in Asian patients with aniridia and one of few similar cases with this kind of genetic mechanism.

Our postulation is based on following evidence: 1) The heterozygous deletion segregated with aniridia in the five affected individuals but not in the unaffected individual, while the exons and splicing regions of *PAX6* are apparently free of mutations. 2) Several publications reported similar observations in patients with aniridia, but the chromosomal breakpoint from the 3′ end of *PAX6* and the fragment of deletion were different. For example, two aniridia pedigrees have been characterized in which the disease segregates with chromosomal re-arrangements that involve 11p13 but do not disrupt the *PAX6* gene since the chromosomal breakpoint is at least 85 kb away from the 3′ end of *PAX6* [8]. Two aniridia pedigrees have also been described in which deletion in the *ELP4* gene region, not involving *PAX6*, was present in all subjects with aniridia but not in the investigated normal relatives [12]. A 1.3 Mb deletion (including seven annotated genes^:^ metallophosphoesterase domain containing 2 (*MPPED2*), doublecortin domain containing 5 (*DCDC5*), *DCDC1*, *DNAJC24, IMMP1L,* zinc finger CLS domain containing 3 (*DPH4*), and *ELP4*) has been characterized that starts 35 kb from the 3′ end of *PAX6* in a patient with aniridia, autism, and mental retardation [11]. Recently a ~406 kb heterozygous genomic deletion containing four annotated genes (*DCDC1, DNAJC24, IMMP1L,* and *ELP4*) was found in patients with aniridia [13]; apparently the gene contents in this deletion are the same as the 566 kb heterozygous deletion in the family we report here. 3) Functional studies in both human cells and animal models confirmed the existence of remote 3′ regulatory elements in the downstream region of *PAX6*. Deletions in this region have been shown to abolish PAX6 expression and cause aniridia and other eye anomalies due to loss of enhancers and a downstream regulatory region [9,19,20].

Little information is known about the four annotated genes within the 566 kb deletion in this study. It is also unknown whether these genes are involved in any of the phenotypic features found in these individuals carrying the deletion. The *DCDC1* gene encodes a member of the doublecortin family, which is highly expressed in testis and fetal brain [21]. *DNAJC24* is one of several enzymes involved in synthesis of diphthamide, which is a unique posttranslationally modified histidine found only in translation elongation factor 2 [22]. *IMMP1L* encodes a peptidase similar to mitochondrial inner membrane peptidase (IMP1), which is one of the catalytic subunits of the IMP complex proteolytically removing the mitochondrial targeting presequence of nuclear-encoded proteins [23]. *ELP4* encodes a component of the six subunit elongator complex, a histone acetyltransferase complex that associates directly with RNA polymerase II during transcriptional elongation. Two recent reports indicate that *ELP4* is possibly associated with the centrotemporal sharp wave electroencephalogram (EEG) trait in rolandic epilepsy and speech sound disorder [24,25].

Submicroscopic copy number variations may play a role in human diseases either by loss of gene expression regulatory elements or by disrupting coding sequences. As well as the point mutations in *PAX6* exons, copy number variation should be investigated in the ﬂanking regions of *PAX6*. We suggest that patients, such as the subjects reported here, should be investigated using high resolution aCGH techniques in a clinical setting if sequencing analyses for *PAX6* in patients with aniridia is negative.

## References

[1] Kokotas H Petersen MB Clinical and molecular aspects of aniridia. Clin Genet 2010 77 409 20 20132240 10.1111/j.1399-0004.2010.01372.x

[2] Hanson IM Fletcher JM Jordan T Brown A Taylor D Adams RJ Punnett HH van Heyningen V Mutations at the PAX6 locus are found in heterogeneous anterior segment malformations including Peters' anomaly. Nat Genet 1994 6 168 73 8162071 10.1038/ng0294-168

[3] François J Lentini F de Rouck F Gillespie's syndrome (incomplete aniridia, cerebellar ataxia and oligophrenia). Ophthalmic Paediatr Genet 1984 4 29 32 6544390 10.3109/13816818409009891

[4] Azuma N Yamaguchi Y Handa H Hayakawa M Kanai A Yamada M Missense mutation in the alternative splice region of the PAX6 gene in eye anomalies. Am J Hum Genet 1999 65 656 63 10441571 10.1086/302529 PMC1377971

[5] Martha A Ferrell RE Mintz-Hittner H Lyons LA Saunders GF Paired box mutations in familial and sporadic aniridia predicts truncated aniridia proteins. Am J Hum Genet 1994 54 801 11 7909985 PMC1918271

[6] Azuma N Hotta Y Tanaka H Yamada M Missense mutations in the PAX6 gene in aniridia. Invest Ophthalmol Vis Sci 1998 39 2524 8 9856761

[7] Xu HE Rould MA Xu W Epstein JA Maas RL Pabo CO Crystal structure of the human Pax6 paired domain-DNA complex reveals specific roles for the linker region and C-terminal subdomain in DNA binding. Genes Dev 1999 13 1263 75 10346815 10.1101/gad.13.10.1263 PMC316729

[8] Fantes J Redeker B Breen M Boyle S Brown J Fletcher J Jones S Bickmore W Fukushima Y Mannens M Danes S Heyningen V Hanson I Aniridia-associated cytogenetic rearrangements suggest that a position effect may cause the mutant phenotype. Hum Mol Genet 1995 4 415 22 7795596 10.1093/hmg/4.3.415

[9] Lauderdale JD Wilensky JS Oliver ER Walton DS Glaser T 3′ deletions cause aniridia by preventing PAX6 gene expression. Proc Natl Acad Sci USA 2000 97 13755 9 11087823 10.1073/pnas.240398797 PMC17648

[10] Crolla JA van Heyningen V Frequent chromosome aberrations revealed by molecular cytogenetic studies in patients with aniridia. Am J Hum Genet 2002 71 1138 49 12386836 10.1086/344396 PMC385089

[11] Davis LK Meyer KJ Rudd DS Librant AL Epping EA Sheffield VC Wassink TH Pax6 3′ deletion results in aniridia, autism and mental retardation. Hum Genet 2008 123 371 8 18322702 10.1007/s00439-008-0484-x PMC2719768

[12] D'Elia AV Pellizzari L Fabbro D Pianta A Divizia MT Rinaldi R Grammatico B Grammatico P Arduino C Damante G A deletion 3′ to the PAX6 gene in familial aniridia cases. Mol Vis 2007 13 1245 50 17679951

[13] Bayrakli F Guney I Bayri Y Ercan-Sencicek AG Ceyhan D Cankaya T Mason C Bilguvar K Bayrakli S Mane SM State MW Gunel M A novel heterozygous deletion within the 3′ region of the PAX6 gene causing isolated aniridia in a large family group. J Clin Neurosci 2009 16 1610 4 19793656 10.1016/j.jocn.2009.03.022

[14] Yuan H Kang Y Shao Z Li Y Yang G Xu N Two novel PAX6 mutations identified in northeastern Chinese patients with aniridia. Mol Vis 2007 13 1555 61 17893655

[15] Yu S Bittel DC Kibiryeva N Zwick DL Cooley LD Validation of the Agilent 244K oligonucleotide array-based comparative genomic hybridization platform for clinical cytogenetic diagnosis. Am J Clin Pathol 2009 132 349 60 19687311 10.1309/AJCP1BOUTWF6ERYS

[16] Yu S Kielt M Stegner AL Kibiryeva N Bittel DC Cooley LD Quantitative real-time polymerase chain reaction for the verification of genomic imbalances detected by microarray-based comparative genomic hybridization. Genet Test Mol Biomarkers 2009 13 751 60 20001581 10.1089/gtmb.2009.0056

[17] Shaikh TH Gai X Perin JC Glessner JT Xie H Murphy K O'Hara R Casalunovo T Conlin LK D'Arcy M Frackelton EC Geiger EA Haldeman-Englert C Imielinski M Kim CE Medne L Annaiah K Bradfield JP Dabaghyan E Eckert A Onyiah CC Ostapenko S Otieno FG Santa E Shaner JL Skraban R Smith RM Elia J Goldmuntz E Spinner NB Zackai EH Chiavacci RM Grundmeier R Rappaport EF Grant SF White PS Hakonarson H High-resolution mapping and analysis of copy number variations in the human genome: a data resource for clinical and research applications. Genome Res 2009 19 1682 90 19592680 10.1101/gr.083501.108 PMC2752118

[18] Itsara A Cooper GM Baker C Girirajan S Li J Absher D Krauss RM Myers RM Ridker PM Chasman DI Mefford H Ying P Nickerson DA Eichler EE Population analysis of large copy number variants and hotspots of human genetic disease. Am J Hum Genet 2009 84 148 61 19166990 10.1016/j.ajhg.2008.12.014 PMC2668011

[19] Griffin C Kleinjan DA Doe B van Heyningen V New 3′ elements control Pax6 expression in the developing pretectum, neural retina and olfactory region. Mech Dev 2002 112 89 100 11850181 10.1016/s0925-4773(01)00646-3

[20] Kleinjan DA Seawright A Mella S Carr CB Tyas DA Simpson TI Mason JO Price DJ van Heyningen V Long-range downstream enhancers are essential for Pax6 expression. Dev Biol 2006 299 563 81 17014839 10.1016/j.ydbio.2006.08.060 PMC2386664

[21] Zeng L Gu S Li Y Zhao E Xu J Ye X Wu Q Wang L Xie Y Mao Y Identification of a novel human doublecortin-domain-containing gene (DCDC1) expressed mainly in testis. J Hum Genet 2003 48 393 6 12820024 10.1007/s10038-003-0033-3

[22] Liu S Milne GT Kuremsky JG Fink GR Leppla SH Identification of the proteins required for biosynthesis of diphthamide, the target of bacterial ADP-ribosylating toxins on translation elongation factor 2. Mol Cell Biol 2004 24 9487 97 15485916 10.1128/MCB.24.21.9487-9497.2004 PMC522255

[23] Burri L Strahm Y Hawkins CJ Gentle IE Puryer MA Verhagen A Callus B Vaux D Lithgow T Mature DIABLO/Smac is produced by the IMP protease complex on the mitochondrial inner membrane. Mol Biol Cell 2005 16 2926 33 15814844 10.1091/mbc.E04-12-1086 PMC1142436

[24] Strug LJ Clarke T Chiang T Chien M Baskurt Z Li W Dorfman R Bali B Wirrell E Kugler SL Mandelbaum DE Wolf SM McGoldrick P Hardison H Novotny EJ Ju J Greenberg DA Russo JJ Pal DK Centrotemporal sharp wave EEG trait in rolandic epilepsy maps to Elongator Protein Complex 4 (ELP4). Eur J Hum Genet 2009 17 1171 81 19172991 10.1038/ejhg.2008.267 PMC2729813

[25] Pal DK Li W Clarke T Lieberman P Strug LJ Pleiotropic effects of the 11p13 locus on developmental verbal dyspraxia and EEG centrotemporal sharp waves. Genes Brain Behav 2010 9 1004 12 20825490 10.1111/j.1601-183X.2010.00648.x

